# Supplementary material for: Methamphetamine Dysregulates Macrophage Functions and Autophagy to Mediate HIV Neuropathogenesis
Source: Biomedicines. 2022 May 27;10(6):1257. doi: 10.3390/biomedicines10061257 (PMC9220012; doi:10.3390/biomedicines10061257)
Supplement: Supplementary file 1 [file biomedicines-10-01257-s001.zip › Supplementary Document.pdf]

Supplementary Material

Table S1. See attached Excel file. Significantly enriched proteins with 24 h meth (50 μM) treatment of HIV-infected macrophages as detected by mass spectrometry, either upregulated or downregulated as indicated. Fold changes of <0.8 (log2(-0.32)) and >1.2 (log2(0.26)) were used as cutoffs for all proteins $p < 0.1$ (log2(3.32)) and $p < 0.5$ (log2(4.32)), $n = 4$.

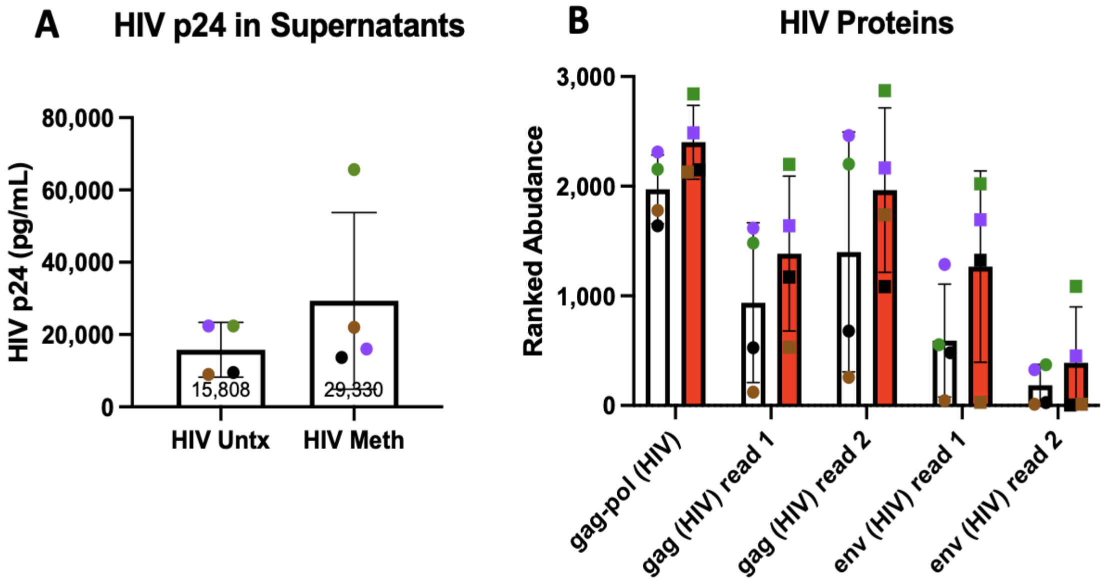

Figure S1. Detected HIV genome proteins (**B**) correlate with p24 alphaLISA data from culture supernatants (**A**). Individual experiments are color coded same as in Figure 4A. HIV Untx (untreated) is in white bars, and HIV Meth is in red bars. No proteins detected were enriched statistically with cutoff of $p < 0.05$. $n = 4$. Error bars represent SD.

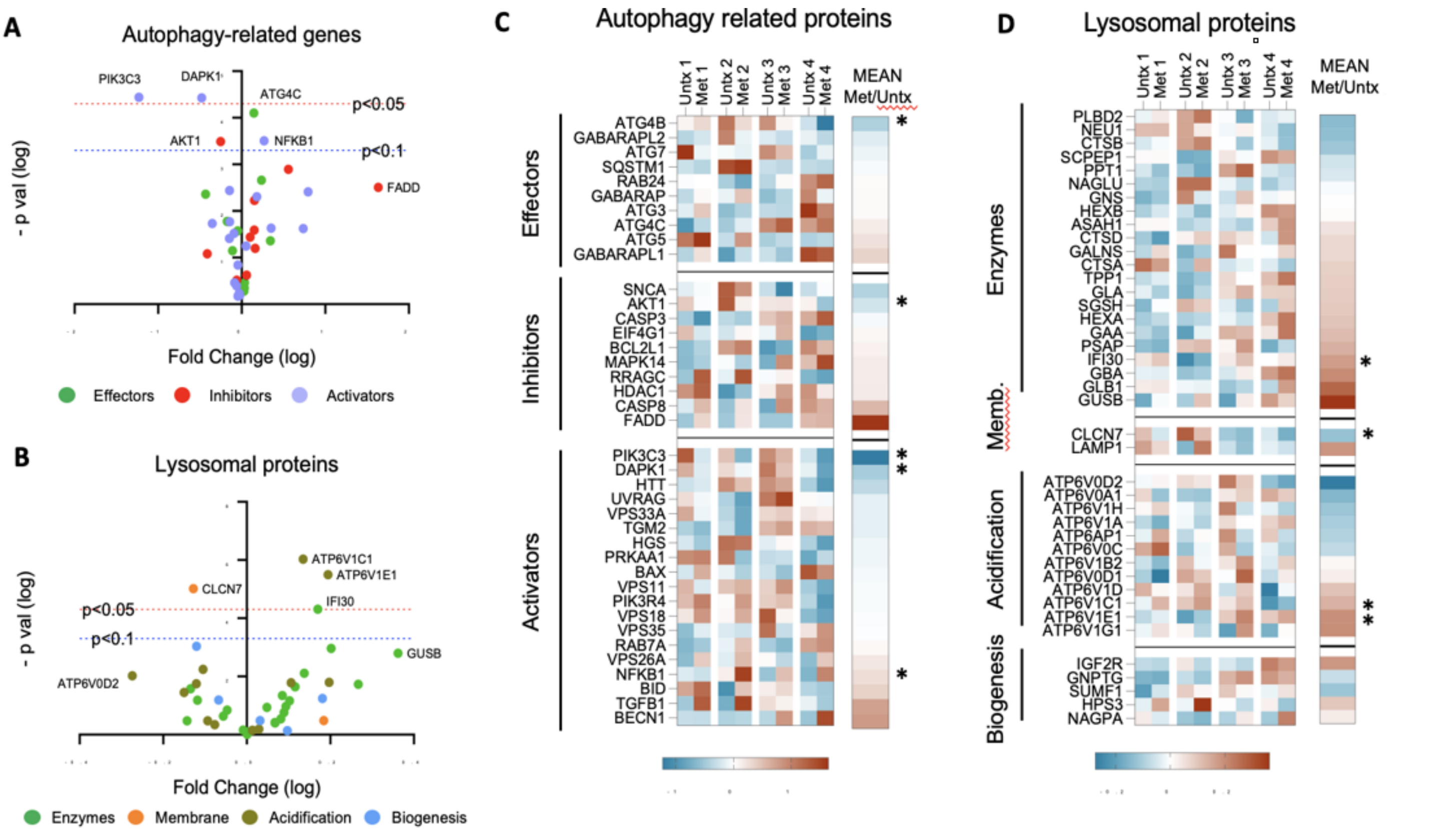

Figure S2. Methamphetamine-induced changes in autophagy/lysosomal components in HIV-infected macrophages**.** Comparative differential proteomics of HIV-infected human macrophages untreated (Untx) or treated with methamphetamine (Met). (**A**,**B**)**.** Log_2 fold change in levels of autophagy (**A**) or lysosome (**B**) related proteins upon treatment with methamphetamine. Names of proteins that significantly increase (right) or decrease (left) upon treatment are shown. Proteins are color-coded based on their functional group as indicated in the legend. Values are paired averages of n = 4 subjects per group. (**C**,**D**) Heat map of levels of autophagy (**C**) and lysosome (**D**) related proteins in HIV-infected macrophages from 4 individual donors untreated or treated with methamphetamine. Average values of the paired changes are shown on the right. *$p < 0.1$, paired t test.

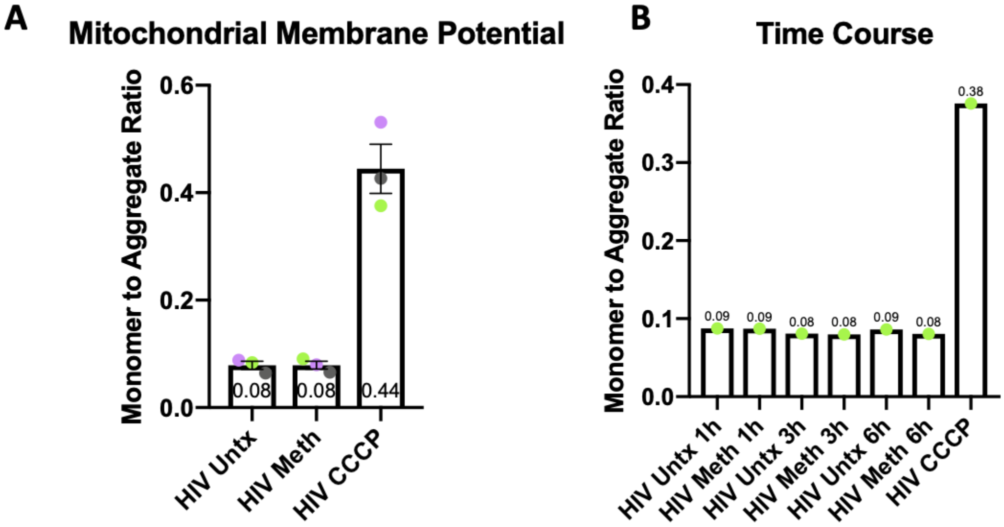

Figure S3. Determination of mitochondrial membrane potential by JC-1 dye fluorometric plate reader assay (Abcam: ab113850, Waltham, MA USA). **(A)** HIV-infected MDM were treated or not (HIV Untx) with meth for 24 h. Another set of cells was treated with 30 μM CCCP for 1 h. In the last 30 min of treatment, 10 μM JC-1 dye was added followed by washing with provided assay buffer. Cells were excited at 475 nm, monomer signal was read at 530 nm, and aggregate signal was read

at 590 nm. Depolarized mitochondria have increased monomer signal and decreased aggregate signal. **(B)** The same assay was repeated but instead meth was added or not for 1 h, 3 h, or 6 h before staining with 10 μM JC-1 dye for 30 min. $n = 1–3$. Error bars represent SEM.

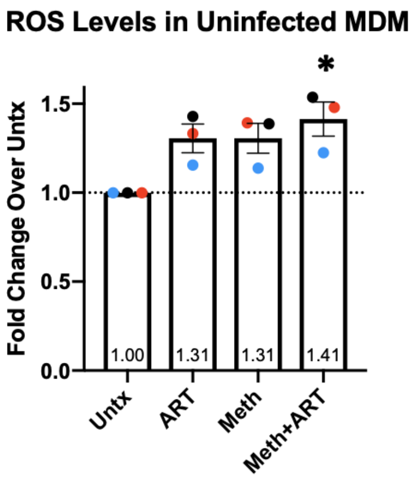

Figure S4. Reactive oxygen species (ROS) levels in uninfected MDM that were untreated (Untx) or treated with meth and/or ART for 24 h. Total ROS were detected by a fluorometric plate reader assay using CM-H2DCFDA staining. $n = 3$, $*p < 0.05$ by one-sample t test.

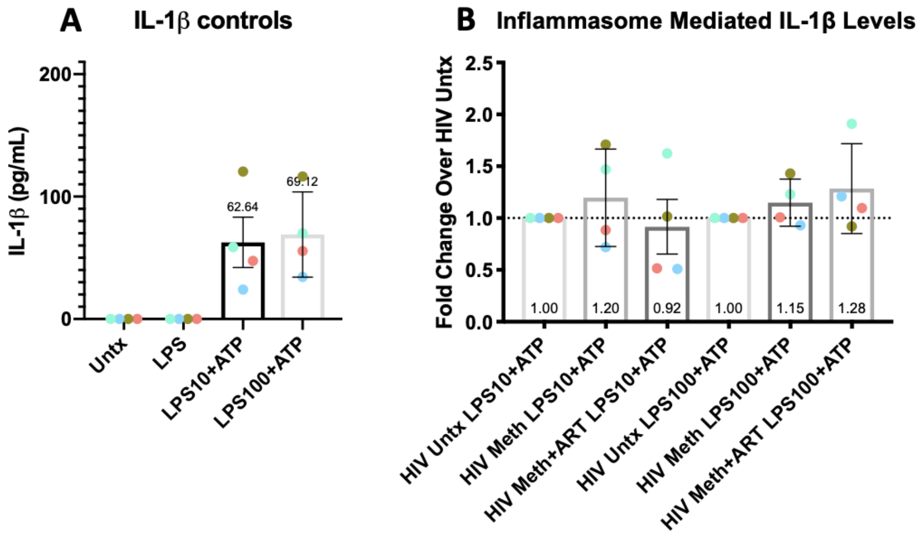

Figure S5. Inflammasome mediated IL-1β release from HIV-infected MDM. Human macrophages were infected with HIV and left untreated (Untx) or treated with meth and/or ART daily for 7 days. Either 10 ng/mL LPS (LPS10) or 100 ng/mL (LPS100) was added to cultures in the last 4 h of the experiment with 5 mM ATP added in the last 30 min (LPS10 + ATP) or (LPS100 + ATP) to stimulate NLRP3 inflammasomes. (**A**) Raw values demonstrating that both LPS and ATP are needed to activate inflammasomes and secrete IL-1β, which was detected in culture supernatants by ELISA (R&D #DY201, Minneapolis, MN USA). (**B**) Fold change in IL-1β levels with either HIV Untx LPS10 + ATP or HIV Untx LPS100 + ATP set to 1.0. $n = 4$. Error bars represent SD for (**A**) and SEM for (**B**).

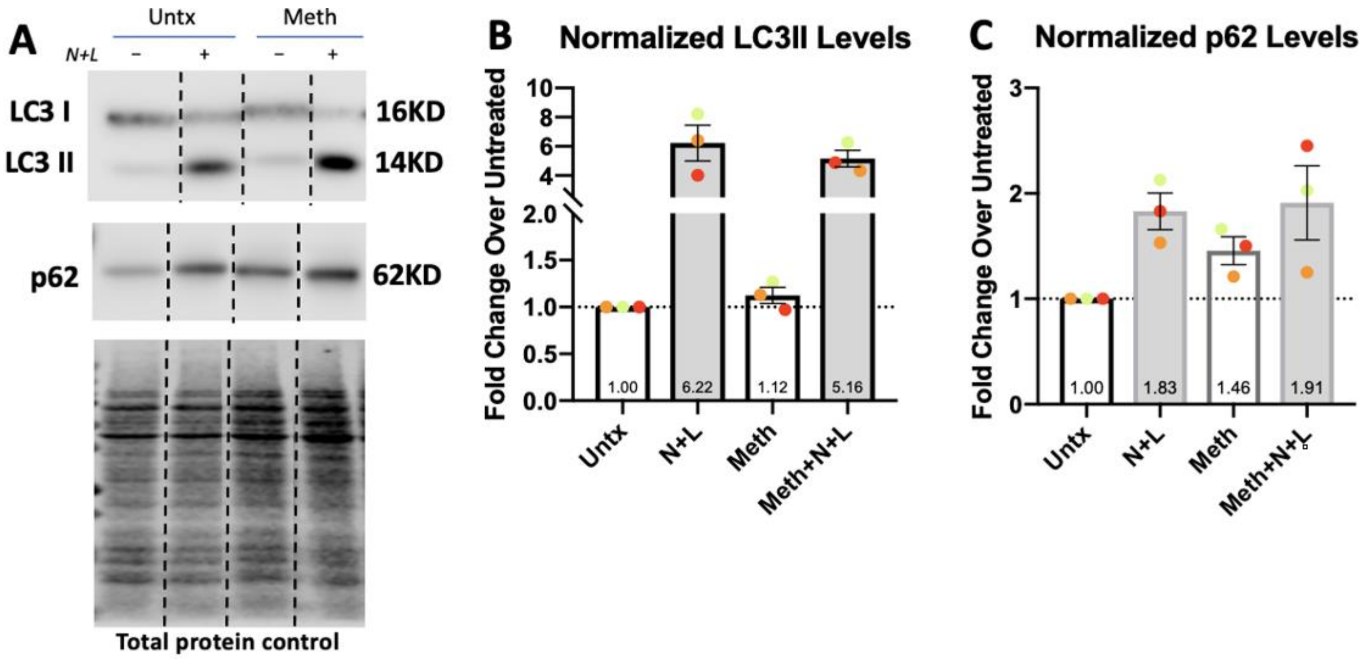

Figure S6. LC3II and p62 Western blot data in uninfected MDM. Western blotting experiments were performed as described in the Materials and Methods section. NH4Cl and leupeptin (N + L) were added to cultures in the last 4 h of treatment with or without meth for 24 h. (**A**) Representative Western blots for LC3 and p62. (**B**) LC3II levels relative to the untreated (Untx) control set to 1.0 in each experiment. (**C**) p62 levels relative to Untx set to 1.0 in each experiment. *n* = 3. Error bars represent SEM.
